# *StMAPKK5* responds to heat stress by regulating potato growth, photosynthesis, and antioxidant defenses

**DOI:** 10.3389/fpls.2024.1392425

**Published:** 2024-05-16

**Authors:** Xi Zhu, Wei Li, Ning Zhang, Hui Jin, Huimin Duan, Zhuo Chen, Shu Chen, Qihua Wang, Jinghua Tang, Jiannan Zhou, Yu Zhang, Huaijun Si

**Affiliations:** ^1^ Key Laboratory of Tropical Fruit Biology, Ministry of Agriculture and Rural Affairs, Zhanjiang, Guangdong, China; ^2^ Key Laboratory of Hainan Province for Postharvest Physiology and Technology of Tropical Horticultural Products, Zhanjiang, Guangdong, China; ^3^ National Key Laboratory for Tropical Crop Breeding, Sanya Research Institute, Chinese Academy of Tropical Agricultural Sciences, Sanya, China; ^4^ State Key Laboratory of Aridland Crop Science, Gansu Agricultural University, Lanzhou, China; ^5^ College of Life Science and Technology, Gansu Agricultural University, Lanzhou, China

**Keywords:** potato, heat stress, StMAPKK5, transpiration, photosynthesis

## Abstract

**Backgrounds:**

As a conserved signaling pathway, mitogen-activated protein kinase (MAPK) cascade regulates cellular signaling in response to abiotic stress. High temperature may contribute to a significant decrease in economic yield. However, research into the expression patterns of *StMAPKK* family genes under high temperature is limited and lacks experimental validation regarding their role in supporting potato plant growth.

**Methods:**

To trigger heat stress responses, potato plants were grown at 35°C. qRT-PCR was conducted to analyze the expression pattern of *StMAPKK* family genes in potato plants. Plant with *StMAPKK5* loss-of-function and gain-of-function were developed. Potato growth and morphological features were assessed through measures of plant height, dry weight, and fresh weight. The antioxidant ability of *StMAPKK5* was indicated by antioxidant enzyme activity and H_2_O_2_ content. Cell membrane integrity and permeability were suggested by relative electrical conductivity (REC), and contents of MDA and proline. Photosynthetic capacity was next determined. Further, mRNA expression of heat stress-responsive genes and antioxidant enzyme genes was examined.

**Results:**

In reaction to heat stress, the expression profiles of *StMAPKK* family genes were changed. The StMAPKK5 protein is located to the nucleus, cytoplasm and cytomembrane, playing a role in controlling the height and weight of potato plants under heat stress conditions. *StMAPKK5* over-expression promoted photosynthesis and maintained cell membrane integrity, while inhibited transpiration and stomatal conductance under heat stress. Overexpression of *StMAPKK5* triggered biochemical defenses in potato plant against heat stress, modulating the levels of H_2_O_2_, MDA and proline, as well as the antioxidant activities of CAT, SOD and POD. Overexpression of *StMAPKK5* elicited genetic responses in potato plants to heat stress, affecting heat stress-responsive genes and genes encoding antioxidant enzymes.

**Conclusion:**

*StMAPKK5* can improve the resilience of potato plants to heat stress-induced damage, offering a promising approach for engineering potatoes with enhanced adaptability to challenging heat stress conditions.

## Highlights

- In response to heat stresses, expression patterns of *StMAPKKs* are altered in different potato tissues;
- *StMAPKK5* influences potato morphological characteristics, including plant weight and height under heat stress conditions;
- *StMAPKK5* regulates the physiological index of potato plants responding to heat stress.
- *StMAPKK5* regulates photosynthesis and transpiration in response to heat stress;
- *StMAPKK5* leads to altered expression of heat stress-responsive genes and antioxidant enzyme genes in potato plants under heat stress conditions.

## Introduction

Over the past century, the Earth’s average surface temperature has been rising (Hansen et al., 2022; Katlane et al., 2023). Evidence indicates that extreme weather events, including heat waves, are increasing in both frequency and intensity (Walsh et al., 2020). Extreme temperatures confer negative roles in photosynthesis, flower pollination, and fruit setting, which leads to lower crop yields and affects food availability (Beillouin et al., 2020; Dalhaus et al., 2020; Nicholson and Egan, 2020; Wi et al., 2020). As the fourth most important crop in agricultural production, potatoes (*Solanum tuberosum* L.) are susceptible to high temperature or adverse water supply (Singh et al., 2020). Due to climate change, tuber growth is inhibited above 33°C and high temperature above 25°C induces leaf senescence (Rykaczewska, 2015). We need to better understand how heat stress affects plant growth and development as global temperatures rise in the future. This could offer valuable insights into breeding programs to improve plant heat-stress tolerance.

Protein kinases (PK) constitute a vast and crucial superfamily of enzymes involved in cellular signaling, regulating a wide array of biological processes (Chen et al., 2021a). The mitogen-activated protein kinases (MAPKs) are important and evolutionarily conserved subfamily of protein kinases that play a central role in transducing signals from the cell surface to the nucleus responding to a variety of stimuli, for instance, heat stress (He et al., 2020; Kumar et al., 2020). The hierarchical organization of MAPK signaling cascades, encompassing the MAPKs, MAPK kinases (MAPKKs or MEKs), and MAPKKs kinases (MAPKKKs or MEKKs), forms a crucial framework for transmitting external signals, amplifying stress signaling, and ultimately facilitating communication between the cellular environment and transcriptional responses (Zwerger and Hirt, 2001; Taj et al., 2010; Zhang and Zhang, 2022). Heat stress can indeed trigger various signaling mechanisms within biological systems in three pathways, including Ca^2+^-dependent salt overlay sensitive signaling and osmotic/oxidative stress signaling (Rodriguez et al., 2010). The role of oxidative stress signaling in promoting osmolyte accumulation through the MAPK cascade, involving G-protein receptors, histidine kinases, and protein tyrosine kinases, aligns with known cellular responses to stress (Ara and Sinha, 2014; Röhm et al., 2021).

Genome-wide analyses have progressively identified multiple *MAPKKs* genes in various plant species. Yin et al. identified 23 *MAPKKs* in the *Gossypiumhirsutum* genome and divided into 4 groups. Most of these genes feature the active site motif-D(I/L)K-, which includes two conserved residues, K (lysine) and D (aspartic acid) (Yin et al., 2021). The cucumber genome contains 6 *MAPKK* genes classified into 4 groups (A-D) (Wang et al., 2015). Ten *MAPKK* genes have been reported in *Arabidopsis* genome, which are also classified into 4 groups (A-D) (Danquah et al., 2014; Choi et al., 2017). Bioinformatic analysis of maize genome revealed 9 *MAPKK* genes and identified 11 subdomains of protein kinases showing serine/threonine specificity (Kong et al., 2013). From the available genome, 5 *MAPKK* genes were identified in tomato (Wu et al., 2014) and 7 in rice (Kumar et al., 2008), respectively. However, systematic studies of the *MAPKK* gene family are still required for potato plant.

The MAPKK factors, as the components of the MAPK signaling pathway, exhibit evolutionary conservation across various organisms and regulate a wide array of functions, such as stomatal development (Bergmann et al., 2004), plant architecture (Yu et al., 2014), chlorophyll synthesis (Li et al., 2022), glucose metabolism (Chardin et al., 2017a), apical meristem (Lee et al., 2019), and flower development (Lafleur et al., 2015). However, studies on how *StMAPKKs* respond to heat stress in potato plants are scarce. This study aimed to describe the expression features of *StMAPKKs* under heat stress situations. Then, our study analyzed the functional aspects of *StMAPKK5*, including its roles in potato plant growth, antioxidation, cell membrane integrity, photosynthesis capacity, and mRNA expression of heat stress-responsive genes and antioxidant enzyme genes. The potato cultivar “Atlantic” is a cultivar introduced and popularised in Guangdong Province. As a high-quality and high-yield potato variety, “Atlantic” has been studied rarely under heat stress. Meanwhile, this study aimed to screen heat tolerance genes, which may provide an effective basis for its molecular breeding for heat stress tolerance.

## Materials and methods

### Plant materials and cultivation

The potato (*Solanum tuberosum* L.) cultivar “Atlantic” was cultivated *in vitro* using Murashige and Skoog (MS) medium with a pH of 5.8–6.0. The medium was supplemented with 8% sucrose and the plants were cultivated for 4 weeks. Next, four-week seedlings were cultured in the dark for 30 days to induce tuber generation. Approximately 1 g of potato tubers with 1 mm germinated buds were transferred into containers (19 cm ×25 cm ×26 cm) filled with soil-vermiculite mixture (1:1, v/v) and cultivated for 5 weeks in Zhanjiang located at latitude 21°11’43’’N and longitude 110°34’56’’E. Soil water holding capacity ranged from 75% to 80%, supplemented with 100 mL of nutrient solution (pH: 5.8; 0.20 mmol/L FeSO_4_, 2.57 mmol/L KH_2_PO_4_, 2.08 mmol/L MgSO_4_, 1.29 mmol/L (NH_4_)_2_SO_4_, and 9.89 mmol/L KNO_3_) every 7 days. Potato plants in each group exhibiting uniform growth appearance were next cultivated under 35°C. Physiological and photosynthetic indexes were assayed at 0 h, 8 h, 12 h, 24 h, and 48 h after cultivation under heat stress conditions. The control plants were cultivated at 22°C. Each group was prepared with three biological replicates and three technical replicates.

To examine the mRNA expression of *MAPKK* family genes in various plant tissues, the potted plants were continually cultured. Flower, root, stem, leaf, petiole, stolon, and shoot were collected at the developmental stage, and tuber was collected at the maturity stage. Next, the plants were cultivated under 35°C, and *StMAPKKs* expression in leaf, stem, and root were examined at 0 h, 1 h, 2 h, 4 h, 8 h, 12 h, 24 h, and 48 h after cultivation under heat stress conditions.

### Construction of transgenic plants

The gene encoding the StMAPKK5 protein was cloned into the pBI121-EGFP plasmid using a previously described method (Li et al., 2020), to develop *StMAPKK5*-overexpressing plants (OE for short). *Agrobacterium tumefaciens* strains LBA4404 was used for transformation experiments. Potato plants low expressing *StMAPKK5* (RNAi or Ri for short) were established with a previously described method (Lu et al., 2019). The primers utilized for plasmid construction are listed in **Table 1.**

**Table 1:** List of specific primers for qRT-PCR and plasmid construction.

Genbank accession	Gene	Forward (5’-3’)	Reverse (5’-3’)	Product length (bp)	Tm
XM_006347752.2	*StEf1α*	GGTTGTATCTCTTCCGATAAAGGC	GGTTGTATCTCTTCCGATAAAGGC	132	60
XM_006363922.2	*StMAPKK1*	TTCCCACGTGCTTTCTCCTC	TCGGCGATCACGAACTAAGG	148	60
NM_001288477.1	*StMAPKK2*	CGATCACAACGGCGAAATCC	CCTCACGCCTGGAGTTGATT	197	60
NM_001318629.1	*StMAPKK3*	TCCAGCTTCTTGACTGCGAG	TGAACAACACCCCCACTTCC	188	60
XM_006353485.2	*StMAPKK4*	CTCGAGTGTGCAACAGGTCA	TGCACAAGGTTCTGGTTGGT	111	60
XM_006351467.2	*StMAPKK5*	GTCAATCTCAAGGGGGAGCC	TTCGTTCCGGCGACATGTAA	118	60
XM_006358116.2	*StFeSOD2*	GCAGCCAAATTCAGCACACT	GGACCAGCTTTCCTCGCTAA	164	60
XM_006350307.2	*StFeSOD3*	TGCTGCCCAGGTATGGAATC	CCTCTCTGCTCAAGACGAGC	194	60
XM_006358693.2	*StMnSOD*	TAGACGTTTGGGAACACGCA	CTCTTCAGGGGCACTCGTTT	129	60
XM_049521383.1	*StCuZnSOD1 *	CCTCCAACAGGTCACTGCTC	TCAGGTCACCCTTGAATGGC	141	60
AF354748	*StCuZnSOD2*	TGTGGCACCATCCTCTTCAC	TCCTGTTGACATGCAGCCAT	138	60
XM_006358985.2	*StPOD12*	CGGCCTTCTTCGTCTTCACT	AAACGACTCTACCGCAGTCC	188	60
XM_006350750.2	*StPOD47*	AGTCTGAGCAGGCCTTTGAC	GCCCATTTTACGCATGGCTT	197	60
XM_006362636.2	*StPOD66*	GCTTTGCCAACAGGGGATTG	TTCAAGCTCGGGTCAGTGTC	139	60
AY442179	*StCAT1*	GCACAGGGATGAGGAGATCG	CTTCTCACGTTTGCCACTGC	106	60
XM_006340770.2	*StCAT2*	GCACAGGGATGAGGAGATCG	CTTCTCACGTTTGCCACTGC	106	60
XM_006341106.1	*StHSFA3*	AGTGCTGGCACCGAGTTATG	GCTGCCTGCAAGGGATCTAT	104	60
XM_006350742.2	*StHSP20-20*	TTTCGGTGATCGACGAAGCA	TTAAGCCCTGGAAGATCGGC	183	60
XM_006360759.2	*StHSP20-33*	TCCAAGCTTCTTCGGTGGTC	CGAGCAGAGGATGGAGCATT	102	60
XM_006345188.2	*StHSP20-44*	GGAGCAATATCGTCGACCCA	AACATGAGCTTCCGGGGTTT	146	60
Z11982.1	*StHSP70*	GTGTTGGTGTATGGCAAAACGA	AGCAACTTGATTCTTGGCTGC	131	60
XM_006362602.2	*StHSP90.2*	AACTCTCCGTTCTTGGAGCG	TTCAATCCCTCCTTGGTGGC	140	60
XM_006352486.1	*StHSP90.4*	TATAGCAGCCGGTGCAGATG	GCTCTCACCAGAAGTGTCCC	178	60
XM_015308529.1	*StP5CS*	CTCAGTCCGTGTGCTTGCTA	AAGAGGCCATTCCCACTTCG	189	60
Subcellular localization		ATGGCTGGACTGGAGGAATTG	TTGAGTAATGAAAAGTTCATGCT		
Overexpression		CTCGAGATGGCTGGACTGGAGGAATTG	GTCGACTTGAGTAATGAAAAGTTCATGCT		
RNA interference		ATGGCTGGACTGGAGGAATTG	AATATGAATTGCTCTCTGAACAAC		

Agrobacterium containing plasmids were cultured for about 48 h in LB medium in addition with 50 mg/L gentamicin and 50 mg/L spectinomycin at 28°C, next harvested by centrifugation (5,000 rpm, 10 min), and re-suspended in MS medium (OD600 = 0.3). The sterile seedling stems (2 cm) were incubated in Agrobacterium suspension for 10 min, and then grown in MS medium (pH: 5.8) containing 7.4 g/L agar, 30 g/L sucrose, 0.5 mg/L 6-BA, 2.0 mg/L ZT, 0.2 mg/L GA3, and 1.0 mg/L IAA and maintained in the dark for 48–72 h. Next, the plants were transferred into differentiation media (MS, 7.4 g/L agar, 30 g/L sucrose, 300 mg/L Timentin, 100 mg/L kan, 0.5 mg/L 6-BA, 2.0 mg/L ZT, 0.2 mg/L GA3, and 1.0 mg/L IAA; PH: 5.8), with media changed every 2 weeks. After induction of adventitious bud, the resistant adventitious buds were transferred to formulated root medium (MS + 7.4 g/L agar+ 30 g/L sucrose + 300 mg/L Timentin + 100 mg/L kan, pH=5.8) until adventitious roots were induced.

### qRT-PCR

TRIzol RNA Extraction kit (Invitrogen, Carlsbad, CA, USA) was used to extract total RNA from the collected samples. The first-strand cDNA of the target genes were synthesized using the First-Strand cDNA Synthesis Kit (TransGen Biotech, Beijing, China). qPCR was conducted using a LightCycler 480 II real-time PCR system (Roche, Rotkreuz, Switzerland) with the reaction mixture comprising 0.8 μL of specific primers (0.5 μM), 100 ng of cDNA (**Table 1**), and 10 μL of SYBR Premix Ex Taq (2 ×) (Takara, Tokyo, Japan). The reactions underwent an initial incubation at 94°C for 3 min, followed by the procedures: 36 cycles of 94°C for 45 s, 59°C for 34 s, and 72°C for 1 min. The relative transcription levels were determined using the 2^-△△Ct^ method.

### Subcellular localization

The expression vector pPBI121-EGFP carrying protein-coding sequence of *StMAPKK5* gene was transformed into *Agrobacterium tumefaciens* GV3101. The primers utilized for plasmid construction are detailed in **Table 1**. Following the method described by Sparkes et al. (2006), tobacco epidermal cells were infiltrated with the transformed strain. A confocal laser scanning microscope (Olympus, Tokyo, Japan) was used to observe the sample. For the autofluorescence of chlorophyll in chloroplasts, the excitation wavelength and transmission range for emission were set at 640 nm and 675 nm, and 488 nm and 510 nm for the GFP-tagged *StMAPKK5* protein. The software Olympus Fluovie was used for imaging processing.

### Phylogenetic analysis and amino acid alignment

MEGA 5.05 software was utilized to construct a phylogenetic tree with a neighbor-joining algorithm. DNAman tool (Lynnon Biosoft, San Ramon, CA, USA) was applied to multiple-sequence alignment of the conserved subdomains.

### Measurements of plant growth

Non-transgenic (NT) and transgenic plantlets, uniformly measuring 2 cm in height, were planted in MS medium under controlled conditions in an incubator with an 8 h-dark cycle and 16 h-photoperiod at 22°C and 3,000 Lx. To induce heat stress, plants were cultivated under 35°C. Four weeks after heat stress treatment, we measured root fresh/dry weight, plant fresh/dry weight, and plant height.

### Measurements of stomatal conductance, transpiration rate, and net photosynthetic rate

Four-week-old potted plants were cultivated under 35°C. After 0 h, 8 h, 16 h, 24 h, and 48 h, we examined stomatal conductance, transpiration rate, and net photosynthetic rate. Stomatal conductance, transpiration rate, and net photosynthetic rate were assessed using a portable LI-6400XT system (Li-COR, Lincoln, NE, USA) between 9:30–11:30. The fourth fully expanded functional leaf was collected from the plant top. The parameters were configured as follows, 50%-70% of relative humidity in leaf chamber, 1,500 μmol·m^-2^·s^-1^ of the photon flux density, and 400 μmol/mol of CO_2_.

### Assays for levels of proline, MDA, and H_2_O_2_, and activities of POD, SOD, and CAT

Four-week-old potted plants were cultivated under 35°C. Following exposure to heat stress for durations of 0 h, 8 h, 16 h, 24 h, and 48 h, the potted plants were assayed for the levels of proline, malondialdehyde (MDA), and hydrogen peroxide (H_2_O_2_), and activities of peroxidase (POD), superoxide dismutase (SOD), and catalase (CAT), superoxide dismutase (SOD), and peroxidase (POD) activities with methodologies previously established in our research (Zhu et al., 2021). Description of detailed experimental procedures are provided in **Supplementary Methods.**

### Assays for relative electrical conductivity and chlorophyll content

Relative electrical conductivity (REC) and chlorophyll content were assayed according to our previous methods (Zhu et al., 2023). Description of detailed experimental procedures are provided in **Supplementary Methods.**

### Statistical analysis

Statistical analysis was performed using GraphPad Prism Software (GraphPad, San Diego, CA, USA) and IBM SPSS 19.0 Statistical Software (IBM, Chicago, IL, USA). Results are presented as mean ± standard deviation. Histograms and line charts were generated using GraphPad Prism software. For multiple comparisons, one-way ANOVA with Tukey test or Dunnett’s T3 for *post-hoc* analysis or two-way ANOVA corrected by Sidak’s multiple comparisons test were employed.

## Results

### Phylogenetic analysis and sequence comparison of StMAPKK proteins

**Figure 1:**
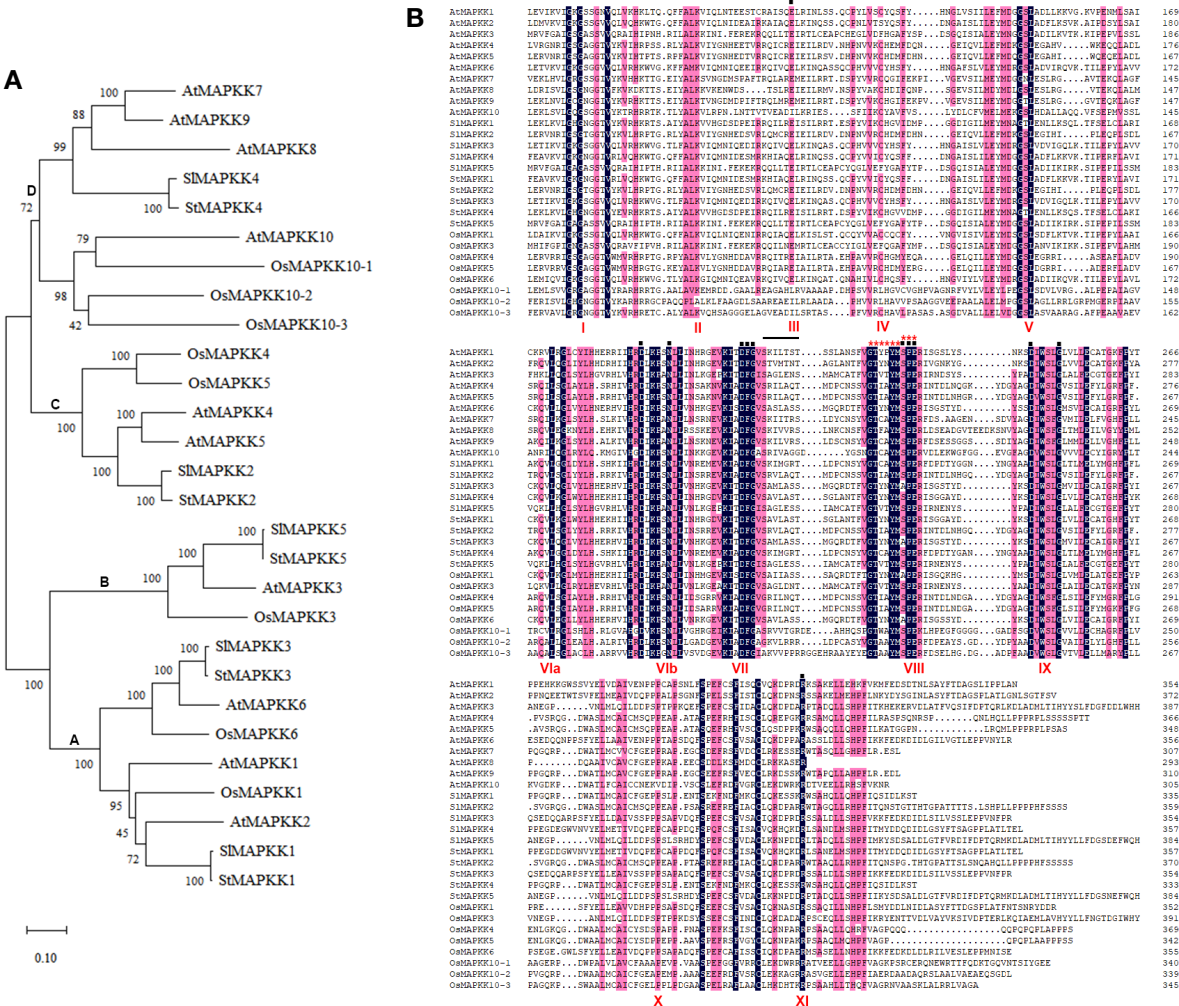
The phylogenetic relationship and protein sequence alignment of MAPKK proteins with MAPKK proteins. **(A)** Dendrogram of MAPKK proteins from *Solanum tuberosum* (St) (StMAPKK1–5), *Arabidopsis thaliana* (At) (AtMAPKK1–10), *Solanum lycopersicum* (Sl) (SlMAPKK1–5), and *Oryza sativa* (Os) (1,3,4,5,6,10–1 and 10–2); Bootstrap values were calculated from 500 resampled datasets with a 50% cut-off; MEGA-X software was used to establish the neighbor-joining phylogenetic tree. **(B)** Protein sequence of MAPKK proteins were aligned using DNAman software; Identical and similar amnio acids were marked in the same colors. The conserved subdomains were indicated on the bottom, using the Roman numbers (I-XI). The dark line marks the active site motif. Red asterisks indicate substrate specificity, and the black dots above the sequences represent activating sites. The conserved consensus motif GXGXXG is marked as I.

After phylogenetic analysis of the amino acid sequences of the reported plant MAPKK homologs, we found StMAPKK5 was homologous with SlMAPKK5, AtMAPKK3 and OsMAPKK3 (**Figure 1A**). There was a high degree of homology between StMAPKK4 and SlMAPKK4, StMAPKK2 and SlMAPKK2, StMAPKK3 and SlMAPKK3, as well as StMAPKK1 and SlMAPKK1. A comparison of the MAPKK proteins from *Solanum tuberosum* (St) (StMAPKK1–5), *Arabidopsis thaliana* (At) (AtMAPKK1–10), *Solanum lycopersicum* (Sl) (SlMAPKK1–5), and *Oryza sativa* (Os) (1, 3, 4, 5, 6, 10–1 and 10–2) showed the presence of the highly conserved domains (**Figure 1B**). StMAPKK5 contains 11 conserved catalytic subdomains typical of MAPKKs and the conserved consensus motif GXGXXG.

### Characterization of *StMAPKK* family genes expression of the various organs of potato

**Figure 2:**
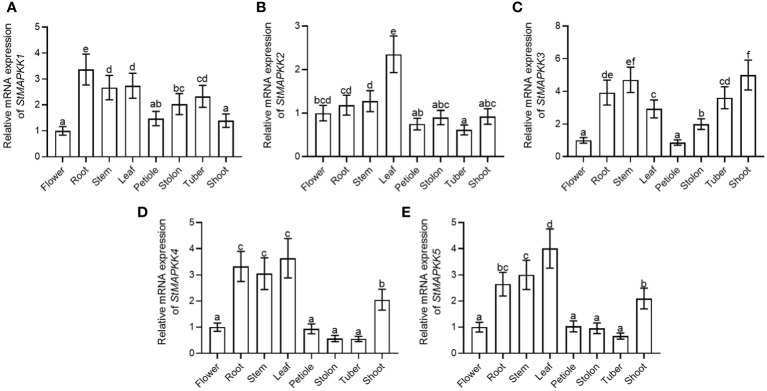
mRNA expression of *StMAPKK* family genes of the various tissues of potato. Relative expression of **(A)** *StMAPKK1*, **(B)** *StMAPKK2*, **(C)** *StMAPKK3*, **(D)** *StMAPKK4*, and **(E)** *StMAPKK5* at mRNA levels, in flower, root, stem, leaf, petiole, stolon, tuber, and shoot. Data were the means ± standard deviation. Different letters indicated significant difference between two groups (*p*-value less than 0.05, calculated by one-way ANOVA, followed by LSD and Duncan or Dunnett’s T3).

A variety of plant tissues (flower, root, stem, leaf, petiole, stolon, tuber, and shoot) were collected to analyze the expression features of *StMAPKK* family genes. *StMAPKK1* showed the highest level of expression in root (**Figure 2A**). *StMAPKK2* displayed the maximum expression in leaf (**Figure 2B**), *StMAPKK3* in stem and shoot (**Figure 2C**), *StMAPKK4* in root (**Figure 2D**), stem and leaf, and *StMAKK5* in leaf (**Figure 2E**). These results represented the tissue-specific expression profiles of *StMAPKK* family genes. We speculated genes within the *StMAPKKs* family may play a crucial role in regulating the growth and development of different tissues and organs in potato.

### Heat stress resulted in differential expression of *StMAPKK* family genes

**Figure 3:**
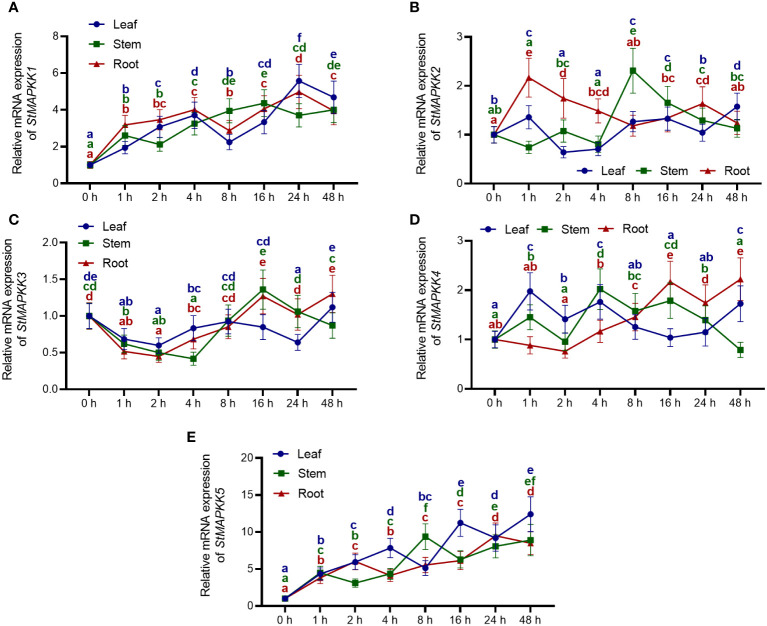
Expression profiles of *StMAPKK* family genes at mRNA level in potato leaves, stem and roots in response to heat stress. Relative mRNA levels of **(A)** *StMAPK1*, **(B)** *StMAPKK2*, **(C)** *StMAPKK3*, **(D)** *StMAPKK4*, and **(E)** *StMAPKK5* in leaves, stem, and roots. Four-week-old normally grown plants were exposed to heat stress (35°C) for 0 h, 1 h, 2 h, 4 h, 8 h, 16 h, 24 h, or 48 h. Data were the means ± standard deviation. Different letters indicated significant difference between two groups (*p*-value less than 0.05, calculated by one-way ANOV, followed by LSD and Duncan or Dunnett’s T3).

Potato leaves, stems and roots were chosen for quantification because the relative expression of *StMAPKK5* gene was found to be higher than in other tissues. At 0 h, 1 h, 2 h, 4 h, 8 h, 16 h, 24 h, or 48 h after exposure to heat stress (35°C), potato leaves, stem and roots were obtained. qRT-PCR was then performed to assay the expression features of *StMAPKK* family genes. The expression of *StMAPKK1* increased steadily with the duration of heat stress (*p* < 0.05) (**Figure 3A**). However, we observed that the expression of *StMAPKK2* (**Figure 3B**), *StMAPKK3* (**Figure 3C**), and *StMAPKK4* (**Figure 3D**) in leaves, stems and roots changed in a disordered manner in response to heat stress (*p* < 0.05). Likewise, we noted that heat stress conditions resulted in a consistent increase in the transcript levels of *StMAPK5* in potato leaves, stems, and roots (*p* < 0.05) (**Figure 3E**). Considering that after treatment, *StMAPKK5* transcript levels were up-regulated at a higher rate than *StMAPKK1–4* (*p* < 0.05). Therefore, we speculated that in response to heat stress, *StMAPKK5* gene may perform crucial molecular functions.

### Impacts of *StMAPKK5* on plant morphological phenotypes under heat stress conditions

**Figure 4:**
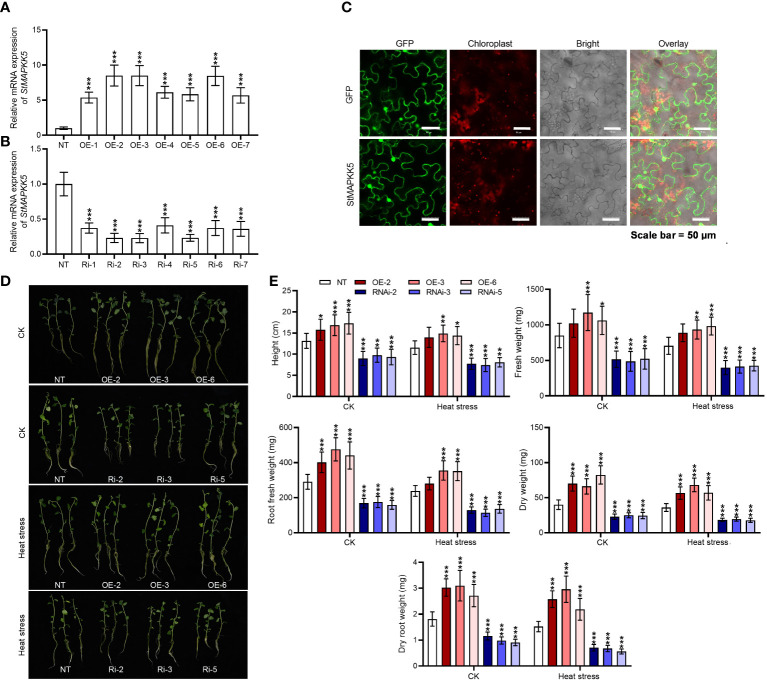
*StMAPKK5* overexpression and under-expression altered the growth of potato plant under heat stress condition. *StMAPKK5* transcription levels in **(A)** *StMAPKK5* overexpression plants and **(B)** *StMAPKK5* under-expression plants. **(C)** Fluorescence images showed *StMAPKK5*-encoding protein located in the nucleus, cytoplasm and cytomembrane; bars = 50 μm; *StMAPKK5* protein was tagged with EGFP (enhanced green fluorescent protein); red fluorescence derives from the chlorophyll. **(D)** Growth morphology of potato plant cultivated under normal and heat stress conditions. **(E)** Plant growth indexes were assayed, including plant height, dry root weight, dry weight, fresh root weight, and fresh weight. Transgenic or non-transgenic plants were measured 0 h, 8 h, 16 h, 24 h, and 48 h after cultivation under normal or heat stress conditions. NT, non-transgenic plants; Ri, pART-StMAPKK5-RNAi-transgenic plants (RNAi-2, RNAi-3, and RNAi-5); OE, pBI121-EGFP-StMAPKK5-transgenic lines (OE-2, OE-3, and OE-6); Data were the means ± standard deviation; p-values (*p < 0.05, **p < 0.01, ***p < 0.001) were calculated by ordinary two-way ANOVA, followed by Tukey’s multiple comparisons test (n = 9).

We generated *StMAPKK5* overexpression plants by introducing pBI121-EGFP-StMAPKK5 and create loss-of-function plants through the induction of pART-StMAPKK5-RNAi. In transgenic plants, *StMAPKK5* mRNA expression was significantly elevated or decreased relative to the wild-type plant (*p* < 0.001) (**Figures 4A, B**). Within the under-expression lines, Ri-2, Ri-3, and Ri-5 were chosen as notable under-expressors, while OE-2, OE-3 and OE-6 were identified as significant over-expressors for subsequent functional analysis under heat stress conditions. In order to better assess the location of *StMAPKK5* protein, we examined by tagging *StMAPKK5* protein with GFP and dissecting the green fluorescence, with the red autofluorescence of chlorophyll as a location reference. The green fluorescence is evenly distributed in the nucleus, cytoplasm and cytomembrane (**Figure 4C**), which was consistent with our previous result (Luo et al., 2024). In comparison with the NT plants, *StMAPKK5* overexpression significantly enhanced the thermotolerance of potato plants as evidenced by the increases in plant height, dry weight, dry root weight, fresh weight, and root fresh weight, under normal and heat stress conditions after 4 weeks of cultivation (*p* < 0.001) (**Figures 4D, E**). In contrast, under normal or heat stress conditions, RNAi-mediated silencing of *StMAPKK5* apparently resulted in the inhibition of plant growth, compared to non-transgenic plants (*p* < 0.001).

### *StMAPKK5* overexpression elevated the ability of ROS scavenging under heat stress condition

**Figure 5:**
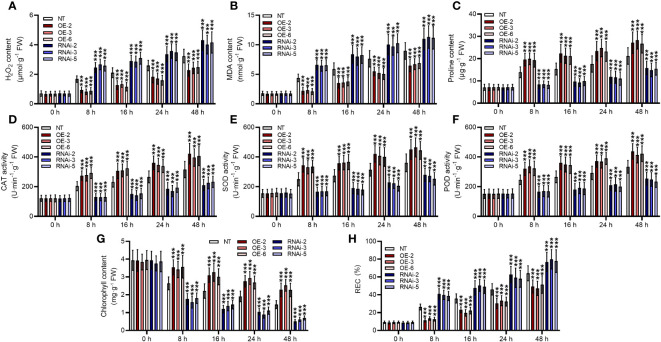
Elevated performance of *StMAPKK5* transgenic plants in scavenging ROS in response to heat stress. **(A)** H_2_O_2_ accumulation, **(B)** MDA content, **(C)** proline content, antioxidant enzyme activity of **(D)** CAT, **(E)** SOD, and **(F)** POD, **(G)** chlorophyll content, and **(H)** REC of potato plant cultivated under normal and heat stress conditions. Transgenic or non-transgenic plants were measured 0 h, 8 h, 16 h, 24 h, and 48 h after cultivated under heat stress conditions. NT, non-transgenic plants; Ri, pART-StMAPKK5-RNAi-transgenic plants (RNAi-2, RNAi-3, and RNAi-5); OE, pBI121-EGFP-StMAPKK5-transgenic lines (OE-2, OE-3, and OE-6); Data were the means ± standard deviation; p-values (***p < 0.001) were calculated by ordinary two-way ANOVA, followed by Tukey’s multiple comparisons test (n = 9).

Heat stresses led to a significant increase in H_2_O_2_ in non-transgenic plants, and it continually increased with prolonged duration of heat stress (*p* < 0.001) (**Figure 5A**). *StMAPKK5* transgenic lines showed significantly reduced H_2_O_2_ production at 8 h, 16 h, 24 h, and 48 h following cultivation under heat stress conditions, compared to non-transgenic plants, while RNAi-mediated silencing of *StMAPKK5* reversely increased the accumulation of H_2_O_2_ (*p* < 0.001). MAD generated in non-transgenic plant was found significantly increased as the extension of heat stress (*p* < 0.001) (**Figure 5B**). Compared to non-transgenic plants, MAD content was decreased in *StMAPKK5* transgenic lines (OE-2, OE-3, and OE-6 after heat stress. In contrast, as compared to non-transgenic plants, knockdown of *StMAPKK5* resulted in the accumulation of MDA under heat stress. The level of proline, as a consequence of heat stress, was increased in non-transgenic plants in a time-dependent manner (**Figure 5C**). The transgenic lines (OE-2, OE-3, and OE-6) accumulated more proline compared to non-transgenic plants under heat stress condition, while *StMAPKK5*-silencing plants were detected with decreased proline content. Under heat stress conditions, the specific activities of CAT, SOD, and POD were consistently higher in non-transgenic plants (*p* < 0.001) (**Figures 5D–F**). After 48 h of heat stress, the activity of CAT, SOD, and POD was found higher in *StMAPKK5*-transgenic lines compared to non-transgenic plants, respectively. However, compared to non-transgenic plants, *StMAPKK5*-silencing plants displayed decreased activities of CAT, SOD, and POD, 48 h after heat stress.

In general, chlorophyll synthesis is sensitive to heat stress, serving as an indicator of the extent of heat stress injury. It was observed that heat stress had an adverse effect on chlorophyll in potato plants (**Figure 5G**). Overexpression of *StMAPKK5* may increase the heat stress tolerance of potato plants by maintaining the total chlorophyll content, compared to non-transgenic plants (*p* < 0.001). *StMAPKK5*-silencing lines appeared to be more susceptible to heat stress, evidenced by a higher reduction in total chlorophyll content than non-transgenic plants (*p* < 0.001). REC have commonly been examined to reflect stress-induced injury of cell membrane. Heat stress significantly exacerbated REC with the extension of time (**Figure 5H**). *StMAPKK5* overexpression profoundly inhibited REC while *StMAPKK5* silence significantly increased REC, compared to the NT plants (*p* < 0.001).

### Overexpression of *StMAPKK5* improved the photosynthetic rate and reduced transpiration responding to heat stresses

**Figure 6:**
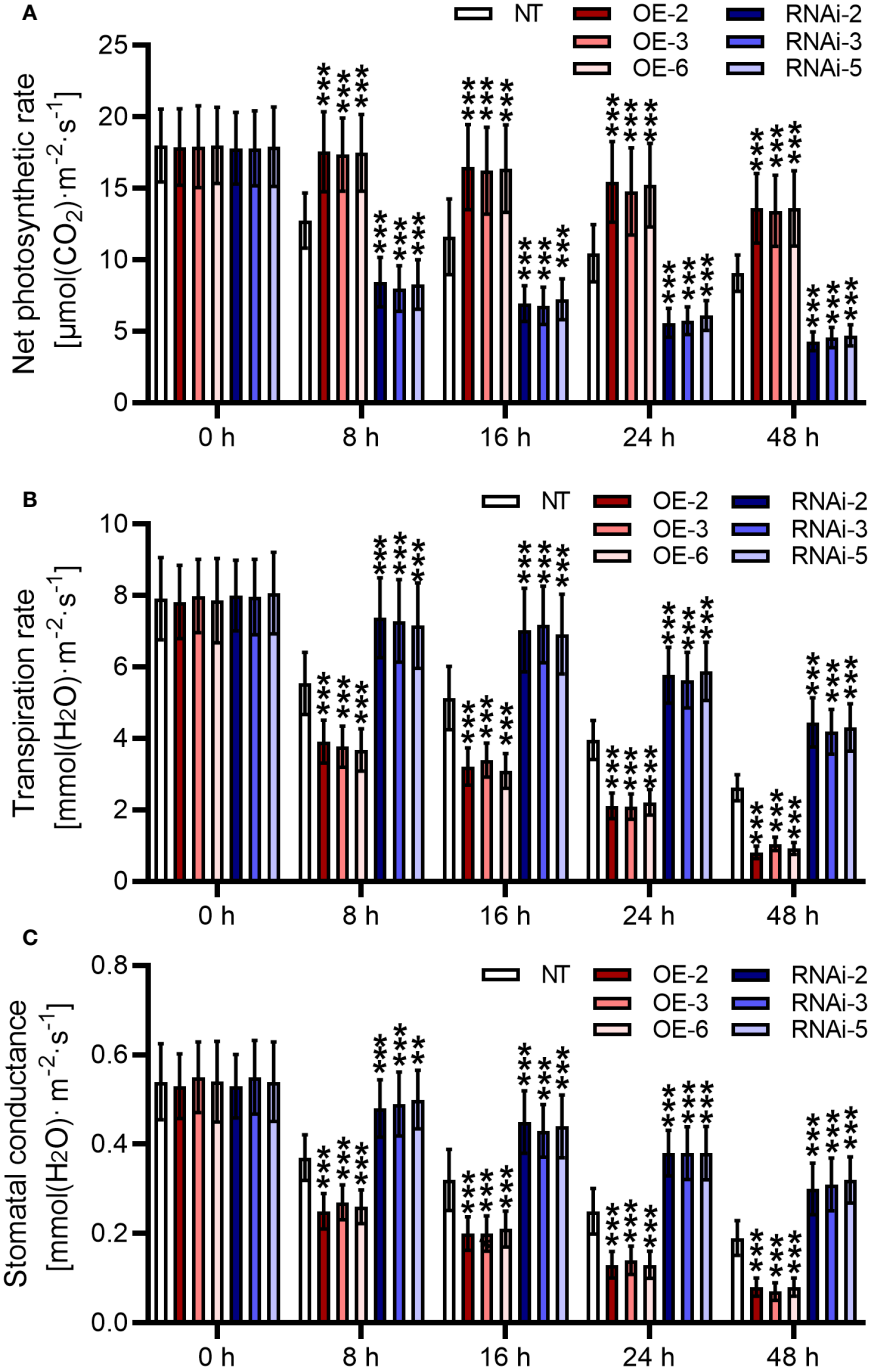
Stomatal apertures and photosynthesis in plants with overexpression or under-expression of *StMAPKK5* responding to heat stresses. **(A)** Net photosynthetic rate, **(B)** transpiration rate and **(C)** stomatal conductance of potato leaves. Transgenic or non-transgenic plants were measured 0 h, 8 h, 16 h, 24 h, and 48 h after cultivated under heat stress conditions. NT, non-transgenic plants; Ri, pART-StMAPKK5-RNAi-transgenic plants (RNAi-2, RNAi-3, and RNAi-5); OE, pBI121-EGFP-StMAPKK5-transgenic lines (OE-2, OE-3, and OE-6). Mean ± standard deviation. p-values (***p < 0.001) were calculated by ordinary two-way ANOVA, followed by Tukey’s multiple comparisons test (n = 9).

Comparisons of measured rates of net photosynthesis suggested that direct suppression of photosynthesis happens at temperatures higher than about 35°C. Cultivation under heat stress contributed to the inhibition of net photosynthetic rate for the NT plants (*p* < 0.05) (**Figure 6A**). However, compared to the NT plants, *StMAPKK5*-overexpressing plants showed increased net photosynthetic rates in response to heat stress (*p* < 0.001). Potato plants lowly expressing *StMAPKK5* failed to maintain net photosynthetic rate in comparison with the NT plants (*p* < 0.001). Transpiration is an essential physiological process, during which course water and mineral nutrients are transferred from soils to plants to dissipate heat. In this study, the transpiration rate of the plants decreased as the duration of thermal incubation increased (**Figure 6B**). *StMAPKK5* overexpression further inhibited transpiration in potato plants, whereas *StMAPKK5* low expression promoted transpiration in instead (*p* < 0.001). Leaf stomata manages plant CO_2_ absorption through transpiration and photosynthesis. It was shown that stomatal conductance was decreased responding to increased temperatures (**Figure 6C**). In transgenic plants, compared to the NT plants, stomatal conductance of *StMAPKK5* overexpression plants decreased markedly (*p* < 0.001). Reversely, *StMAPKK5* silence maintained the stomatal conductance relative to the NT plants (*p* < 0.001). These results indicated that in response to heat stresses, overexpression of *StMAPKK5* increased photosynthetic rate and decreased transpiration and stomatal conductance.

### StMAPKK5 participated in regulating heat-responsive gene expression

**Figure 7:**
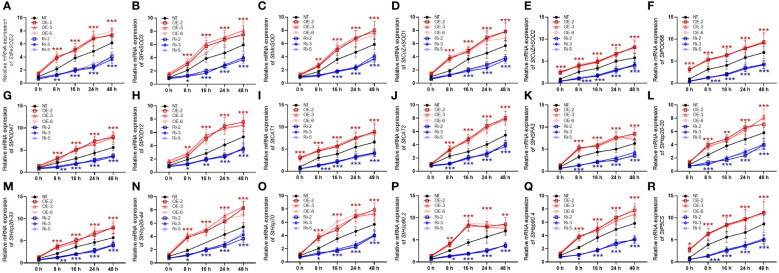
Alterations in mRNA expression of antioxidant enzyme genes and heat-stress responsive genes in plants with overexpression or under-expression of *StMAPKK5* under heat stress conditions. mRNA expression of **(A)** *StFeSOD2*, **(B)** *StFeSOD2*, **(C)** *StPOD*, **(D)** *StCuZnSOD1*, **(E)** *StCuZnSOD2*, **(F)** *StPOD66*, **(G)** *StPOD47*, **(H)** *StPOD12*, **(I)** *StCAT1*, **(J)** *StCAT2*, **(K)** *StHSFA3*, **(L)** *StHsp20–20*, **(M)** *StHsp20–33*, **(N)** *StHsp20–44*, **(O)** *StHsp70*, **(P)** *StHsp90.2*, **(Q)** *StHsp90.4*, and **(R)** *StP5CS* in potato leaves were estimated 0 h, 8 h, 16 h, 24 h, and 48 h after cultivation under 35°C. NT, non-transgenic plants; Ri, pART-StMAPKK5-RNAi-transgenic plants (RNAi-2, RNAi-3, and RNAi-5); OE, pBI121-EGFP-StMAPKK5-transgenic lines (OE-2, OE-3, and OE-6). Mean ± standard deviation. p-values (*p < 0.05, **p < 0.01, ***p < 0.001) were calculated by ordinary two-way ANOVA, followed by Tukey’s multiple comparisons test (n = 9).

To protect the structure and operation of plant cells from the damaging effects of oxidative stress caused by heat stress, the antioxidant defense system is generated, consisting of antioxidant enzymes, like FeSOD, MnSOD, and CuZnSOD, in chloroplasts, mitochondria, and peroxisomes. Under heat stress conditions, NT plants showed a significant increase in mRNA expression of *StFeSOD2*, *StFeSOD*, *StMnSOD*, *StCuZnSOD1*, and *StCuZnSOD2* (**Figures 7A–E**). *StMAPKK5*-transgenic plants (OE-2, OE-3, and OE-6 lines) exhibited increased sensitivity to heat stress, suggesting a higher mRNA expression of *StFeSOD2*, *StFeSOD3*, *StMnSOD*, *StCuZnSOD1* and *StCuZnSOD2*, while Ri-2, Ri-3, and Ri-5 plants exhibited a decreased expressed compared to the NT plants, after 48 h of cultivation under heat stress condition (*p* < 0.001). However, *StMAPKK5*-low expressing plants showed decreased gene expression (*p* < 0.001). Peroxidases, encoded by *StPOD66*, *StPOD47*, and *StPOD12* are enzymes involved in scavenging ROS, in response to heat stress. The *CAT* genes encode for catalase, such as *StCAT1* and *StCAT2*, playing a vital role in the antioxidant defense system by scavenging ROS generated under heat stress situations. In the NT plants, *StPOD66* (**Figure 7F**), *StPOD47* (**Figure 7G**), *StPOD1* (**Figure 7H**), *StCAT1* (**Figure 7I**), and *StCAT2* (**Figure 7J**) was highly expressed in response to heat stress (*p* < 0.001). Compared to the NT plants, the transgenic potato lines (OE-2, OE-3, and OE-6) increased the gene expression, while the RNAi plants profoundly decreased the mRNA expression of peroxidases, in response to heat stress (*p* < 0.001). The *StHSFA3*-encoded protein belongs to the plant heat shock transcription factor family, orchestrating the transcription of genes that encode heat shock proteins (HSP) assisting the plant in coping with heat-induced damage (Friedrich et al., 2021). After 48 h of cultivation under heat stress condition, the NT plants displayed increased mRNA expression of *StHSFA3* (**Figure 7K**), *StHsp20–20* (**Figure 7L**), *StHsp20–33* (**Figure 7M**), *StHsp20–44* (**Figure 7N**), *StHsp70* (**Figure 7O**), *StHsp90.2* (**Figure 7P**), and *StHsp90.4* (**Figure 7Q**). In contrast to the NT plants, the OE lines showed enhanced transcriptional levels, while the Ri plants exhibited decreased expression of the genes, under heat stress conditions (*p* < 0.001). The *StP5CS* gene, which encodes △1-pyrroline-5-carboxylate synthetase, plays a critical role in proline biosynthesis, and proline is an important amino acid involved in maintaining cellular osmotic balance and scavenging ROS. Consistently, the NT plants increased the mRNA expression of *StP5CS*, 48 h after cultivation under heat stress conditions (*p* < 0.001) (**Figure 7R**). In comparison with the NT plants, the mRNA expression of *StP5CS* was evidently enhanced in the OE lines, while reduced in the Ri plants, in response to heat stress. Our results suggested that overexpression of *StMAPKK5* triggered genetic responses of potato plant to combat heat stress.

## Discussion

The effects of high temperatures are now evident in the reduced yield, nutrient depletion, and diminished quality of potatoes in an aberrant environment (Lee et al., 2020; Singh et al., 2020). The MAPK cascade is a crucial signaling pathway that plays a key module in transducing signals from environmental factors such as heat. The cascade has been extensively investigated and detailed in model plants like Arabidopsis, and crops such as rice owing to their relatively well-established research resources (Kumar et al., 2020; Chen et al., 2021). However, understanding the MAPK cascade in response to heat stress has been more challenging due to the complexity of its genome. Here, transgenic potato plants overexpressing *StMAPKK5* displayed an improved heat stress tolerance in comparison to the non-transformed controls. Overexpression of *StMAPKK5* triggered antioxidant enzyme genes, such as *StFeSOD2*, which was then implicated in regulating potato growth and physiology. As a result, *StMAPKK5* can be used to create varieties that are more resistant to heat stress.

Numerous MAPKKs have been detected in a variety of plant species, including *Arabidopsisthaliana* (MAPK group, 2002), *Populus trichocarpa* (Nicole et al., 2006), *Capsicum annuum* (Liu et al., 2015), *Zea mays* (Kong et al., 2013), and *Oryza sativa* (Kumar et al., 2008). We analyzed the phylogenetic relationship of MAPKK proteins from *Solanum tuberosum*, *Arabidopsis thaliana*, *Solanum lycopersicum*, and *Oryza sativa*, and performed multiple alignment, to provide a systematic phylogenetic analysis of MAPKK proteins. Plant MAPKKs feature the phosphorylation site motif S/T-X_5_-S/T and a presumptive MAPK-docking domain characterized by the sequence K/R-K/R-K/R-X_1–6_-LX-L/V/I (Chae et al., 2010). In StMAPKK1, StMAPKK2, StMAPKK3, StMAPKK4, and StMAPKK5, we found S/G-X5-S/T/G/R motif, while K/R-K/R-K/R-X1–6-LX-L/V/I was not noted. Sequence alignment placed the potato MAPKKs into four groups, A (StMAPKK1 and StMAPKK3), B (StMAPKK5), C (StMAPKK2), and D (StMAPKK4).

The modification of MAPKK protein expression under abiotic and biotic stress conditions appear to be a plant strategy for adapting and defending against challenging survival environments. Yang et al. revealed that in response to heat stress, the transcription level of *OsMKK1* in *Xanthomonas oryzae* was apparently up-regulated (Yang et al., 2024). As for the abiotic stress, Kumar et al. *OsMAPKK1*, *OsMAPKK3*, *OsMAPKK4*, *OsMAPKK6*, and *OsMAPKK10–2* expression levels are differentially regulated by heat, cold, drought, and salinity stresses (Kumar et al., 2008). In *Prunus mume* under exposure to cold, the expression of 4 *PmMKK* genes (*PmMKK5*, *PmMKK6*, *PmMKK20*, and *PmMKK3*) is reduced with prolonged treatment (Wen et al., 2023). The expression patterns of *MKK* genes were determined in different tissues of poplar by Wang et al., and the expression of *MKK2a* was altered after cultivation under salt tress (Wang et al., 2022). However, limited studies have explored the expression patterns of *StMAPKKs* under heat stress conditions. Considering that plant MAPKKs have crucial functions responding to abiotic stresses, and their expressions are regulated by heat stress (Kumar et al., 2008; Kumar and Sinha, 2013), we firstly investigated *StMAPKKs* expression distribution in different organs of potato plant and expression profile under heat stress conditions. The expression of *StMAPKK1*, *StMAPKK2*, *StMAPKK3*, *StMAPKK4*, and *StMAPKK5* was distinctly distributed in flower, root, stem, leaf, petiole, stolon, tuber, and shoot. Furthermore, mRNA expression of the five genes in leaf, stem and root were affected to different extent. It is noteworthy that the increase in *StMAPKK5* expression was relatively high and stable. Subsequently, we further constructed *StMAPKK5* overexpression and low-expression plants to analyze its biological functions.

It’s concerning to see the visible impact of aberrant environmental conditions on potato production, nutrient levels, and quality. Transgenic technology has been explored in creating varieties with traits like resistance to tolerance to environmental stressors like high temperature. Chen et al. concluded that the interaction between SaMKK2 and SaMAPK4/7 positively induces the expression of the downstream genes (*SLD2*, *OPR2*, and *CBFs*), thereby endowing potatoes with innate cold resistance (Chen et al., 2022). *StMKK1* is involved in potato defense against the potato late-blight pathogen *Phytophthora infestans*, the gray-mold fungal pathogen *Botrytis cinerea*, and the bacterial wilt pathogen *Ralstonia solanacearum* (Chen et al., 2021b). However, it is still unknown whether *StMAPKK5* mediates the growth of potato plant under heat stress. Our results showed that in response to heat stress, *StMAPKK5* overexpression maintained or even fostered the plant growth, while *StMAPKK5* down-regulation even decreased the growth compared to the non-transgenic plants. Duan et al. suggested a potential relationship between *OsMKK4* and grain size or growth (Duan et al., 2014). *GhMKK3* overexpression facilitates root growth in transgenic *N. benthamiana* (Wang et al., 2016). These findings are consistent with our results, providing a rationale for the involvement of *MAPKK* genes in plant growth.

Plants activate various defense mechanisms to alleviate the harmful impacts of oxidative stress or consequential injury to cells or tissues in response to abiotic stresses (drought, cold, heat, and salinity stresses) (Sachdev et al., 2021). These defense mechanisms include various non-enzymatic antioxidants and antioxidant enzymes (SOD, CAT, and POD) to neutralize ROS and maintain cellular homeostasis (Ahmad et al., 2009; Caverzan et al., 2016). High temperature affects multiple cellular processes, including photosynthesis, respiration and protein stability, and the disruption of these normal physiological activities leads to an overproduction of ROS and causes oxidative stress (Kumar, 2012; Posch et al., 2019; Zahra et al., 2023). Previous work has reported that in response to heat stress, MAPK cascade is involved in signal transduction, which affects plant physiological processes, such as photosynthesis, respiration, transpiration, nutrient uptake, tropisms, and senescence (Lee et al., 2016; Chardin et al., 2017b; Gouda et al., 2020; Liu et al., 2020). In this study, there was a reduction in contents of H_2_O_2_ and MDA, and an increase in antioxidant activities, induced by *StMAPKK5* overexpression under heat stress conditions. Chlorophyll content and REC are regarded as the physiological parameters mostly affected by heat and drought stress (Rehman et al., 2016). From our findings, it can be concluded that *StMAPKK5* overexpression can alleviate the adverse effects on photosynthetic efficiency and cell membrane integrity.

SOD functions in the primary defense against oxidative damage in cells, and participates in the detoxification, neutralization, scavenging, and converting of superoxide radicals (Alscher et al., 2002; Mishra and Sharma, 2019). There are three main types of SOD, each containing a different metal cofactor, including FeSOD, MnSOD, and CuZnSOD (Mishra and Sharma, 2019). Catalase detoxifies hydrogen peroxide and maintains redox homeostasis responding to heat stress (Dat et al., 1998). Peroxidase prevents the accumulation of hydrogen peroxide and reduce oxidative stress, working in coordination with other antioxidants like SOD and catalase (Veal and Day, 2011). *StMAPKK5* overexpression significantly enhances the mRNA expression of SOD, catalase and peroxidase responding to heat stress. HSFA protein activate HSP expression, which are molecular chaperones that assist in protein refolding and prevent protein denaturation under heat stress conditions (Bourgine and Guihur, 2021; Khan et al., 2021). From our results, the increase in *StHSFA3* mRNA expression and the induction of *StHsp20–20*, *StHsp20–33*, *StHsp20–44*, *StHsp70*, *StHsp90.2*, and *StHsp90.4* seems to be related to *StMAPKK5* overexpression. P5CS gene is involved in the biosynthesis of proline that serves as an osmoprotectant, maintaining osmotic balance during heat stress (Liu and Wang, 2020). *StMAPKK5* overexpression-induced heat stress resistance may be through the regulation of StP5CS gene. Thus, *StMAPKK5* overexpression modulated relative expression of heat-stress responsive genes in response to heat stress.

## Conclusions

Extreme high temperature leads to lower crop yields and confers negative roles in food availability. Here, we found the constructed *StMAPKK5*-transgenic potato plants exhibited an intensified heat stress tolerance compared to the non-transgenic plants. However, *StMAPKK5* deficiency showed relatively poor thermal stress resistance. The adverse effects of heat stress on antioxidant activities POD, CAT, and SOD, photosynthetic efficiency and cell membrane integrity were alleviated by *StMAPKK5* overexpression. The enhanced heat stress resistance in plants due to *StMAPKK5* overexpression may be attributed to alterations in the relative expression of antioxidant enzyme genes and heat-stress responsive genes.

## Data availability statement

The original contributions presented in the study are included in the article/**Supplementary Material**, further inquiries can be directed to the corresponding author/s. The MAPKK protein sequences discussed in this article can be accessed at the National Center for Biotechnology Information (NCBI) website (https://www.ncbi.nlm.nih.gov/) under the specified accession number: AtMKK1 (NP_194337.1), AtMKK2 (NP_001031751.1), AtMKK3 (NP_001318713.1), AtMKK4 (NP_175577.1), AtMKK5 (NP_001319606.1), AtMKK6 (NP_200469.1), AtMKK7 (NP_173271.1), AtMKK8 (NP_187274.1), AtMKK9 (NP_177492.1), AtMKK10 (NP_174510.1), SlMAPKK1 (NP_001304158.1), SlMAPKK2 (NP_001234588.1), SlMAPKK3 (NP_001234591.1), SlMAPKK4 (NP_001234595.1), SlMAPKK5 (XP_010317547.1), StMAPKK1 (XP_006353547.1), StMAPKK2 (NP_001275406.1), StMAPKK3 (NP_001305558.1), StMAPKK4 (XP_006363984.1), StMAPKK5 (XP_006351529.1), OsMAPKK1 (XP_015644525.1), OsMAPKK3 (XP_015643944.1), OsMAPKK4 (XP_015627603.1), OsMAPKK5 (XP_015643444.1), OsMAPKK6 (XP_015621394.1), OsMAPKK10–1 (XP_015624529.2), OsMAPKK10–2 (XP_015628376. 1), OsMAPKK10–3 (XP_015629634.1).

## Author contributions

XZ: Writing – original draft, Writing – review & editing. WL: Writing – original draft, Writing – review & editing. NZ: Writing – original draft, Writing – review & editing. HJ: Writing – original draft, Writing – review & editing. HD: Writing – original draft, Writing – review & editing. ZC: Writing – original draft, Writing – review & editing. SC: Writing – original draft, Writing – review & editing. QW: Writing – original draft, Writing – review & editing. JT: Writing – original draft, Writing – review & editing. JZ: Writing – original draft, Writing – review & editing. YZ: Writing – original draft, Writing – review & editing. HS: Writing – original draft, Writing – review & editing.
