# Supplementary material for: StMAPKK5 responds to heat stress by regulating potato growth, photosynthesis, and antioxidant defenses
Source: Front Plant Sci. 2024 May 16;15:1392425. doi: 10.3389/fpls.2024.1392425 (PMC11137293; doi:10.3389/fpls.2024.1392425)
Supplement: Supplementary file 1 [file Table_1.docx]

**SUPPLEMENTARY METHODS**

**Proline content**

Proline content was assayed based on a previous method (Bates et al., 1973) with modification. In short, approximately 0.5 g leaves were homogenized in 10 mL of 3% sulfosalicylic acid, and the mixture was filtered through Whatman filter paper. The filtrate (2 mL) was incubated with 2 mL acid-ninhydrin and 2 mL of glacial acetic acid at 100^o^C for 30 min. Then the mixture was extracted with 4 mL toluene, and the absorbance was detected at 520 nm using a TU-1810 ultraviolet spectrophotometer (Purkinje, Beijing, China).

**MDA content**

MDA content was examined using a well-established method reported by Heath and Packer (Heath and Packer 1968) with modification. Briefly, 1 g of leaves was homogenized in Tris–HCl buffer (50 mM, pH 8.0) for 30 s using BILON-J1500 blender (Waring, New Hartford, Conn., USA). The suspension was filtered through four layers of cotton cloth and then centrifuged for 1 min (200× g). The supernatant was centrifuged at 200× g for 10 min, and the chloroplast precipitate was collected and incubated in a 10 mL tube containing Tris–HCl. An equal aliquot of 0.5% thiobarbituric acid (TBA) in 20% trichloroacetic acid was mixed with the extract. The mixture was heated for 25 min at 95^o^C. The supernatant was collected by centrifugation and assayed at 530 nm for the absorbance.

**H_2_O_2_ content**

H_2_O_2_ was examined according to a previous method reported by Bouaziz et al. (Bouaziz et al. 2015) with modification. Briefly, the leaves (1 g) were digested in 2 mL of 0.1% trichloroacetic acid, followed by centrifugation (12,879.36 × g, 15 min). Then, 0.5 mL of supernatant was incubated with 0.5 mL of potassium phosphate (10 mM, pH 7.0) and 1 mL of potassium iodide (1 mol/L). The absorbance was examined at 390 nm.

**POD activity**

For POD activity, 5.0 g leaves were homogenized in 10 mL of phosphate buffered saline. Then the supernatant was collected by centrifugation (3000× g, 10 min), transferred into a 25-mL volume tric flask and diluted with phosphate buffered saline. Then 0.1 mL extracted solution was incubated with the reactant system containing 2.9 mL of 0.05 mol/L phosphate buffered saline, 1.0 mL of 2% H_2_O_2_ and 1.0 mL of 0.05 mol/L guaiacol at 37^o^C for 15 min. The reaction was terminated by adding 2.0 mL of TCA. The reaction mixture was filtered, and then the absorbance was detected at 470 nm. The extracted solution boiled for 5 min was used as a control.

**SOD activity**

For SOD activity, 0.5 leaves were homogenized in phosphate buffered saline and 5 mL mixture was obtained. The supernatant was collected by centrifugation at 155.4 × g for 20 min. The extract (0.05 mL) was incubated with the chromogenic reagent consisting of 1.5 mL of 0.05 mol/L phosphate buffered saline, 0.3 mL of 130 mM methionine, 0.3 mL of 750 μM nitroblue tetrazolium, 0.3 mL of 100 μM EDTA-Na_2_, 0.3 mL of 20 μM riboflavin, and 0.25 mL H2O under 4000 Lux for 20 min. The mixture was maintained in the dark as the control. The

absorbance was examined at 560 nm.

**CAT activity**

For CAT activity, 2.5 g of leaves were homogenized in 25 mL of phosphate buffered saline (pH 7.8), and the supernatant was collected by centrifugation at 2486.4 × g for 15 min, followed by incubation with 2.5 mL of 0.1 M H_2_O_2_ at 30^o^C for 10 min. The reaction was terminated by adding 2.5 mL of 10% H_2_SO_4_. The content of CAT was examined by 0.1 M KMnO_4_ titration in the presence of H_2_SO_4_. The extracted solution boiled for 5 min was used as a control.

**Relative electrical conductivity**

Relative electrical conductivity (REC) was examined using a conductivity meter according to a method reported by Yu (Yu et al., 2006) with modification. In short, 0.2 g leaves were cut into 1 cm slices and transferred to a centrifuge tube containing 5 mL deionized water. The tubes were incubated in a SHA-C water-bathing constant temperature vibrator (Yineng, Changzhou, China) and shaken at 100 rpm at 25^o^C for 2 h. The electrical conductivity (C1) was measured using DDS-IIA conductivity meter (Lei-ci, Shanghai, China). Then the samples were boiled for 10 min, followed by being assayed for the electrical conductivity (C2). REC was calculated according to the formula: REC (%) = C1 / C2 × 100%.

**Chlorophyll content**

Chlorophyll content in potato leaves was examined using the commercial chlorophyll assay 165 kit according to the manuscript’s instruction (Item No. Cat#BC0990; Solarbio, Beijing, China). Fresh potato leaves were collected and washed in distilled water. After draining the surface of the leaves, the midrib of the leaves was removed and cut into pieces. Approximately 0.1 g of leaves were weighted and ground thoroughly in 1 mL water in the dark, which were then entirely transferred into a 10-mL volumetric flsk, diluted with water to volume, and mixed. The volumetric flsk was maintained in the dark for 3 h. The absorbance of the supernatant was measured at a wavelength of 663 nm and 645 nm using a spectrophotometer model (Perkin Elmer, Shelton, CT, USA).
