# Supplementary figures and images for: StMAPKK5 responds to heat stress by regulating potato growth, photosynthesis, and antioxidant defenses
Source: Front Plant Sci. 2024 May 16;15:1392425. doi: 10.3389/fpls.2024.1392425 (PMC11137293; doi:10.3389/fpls.2024.1392425)

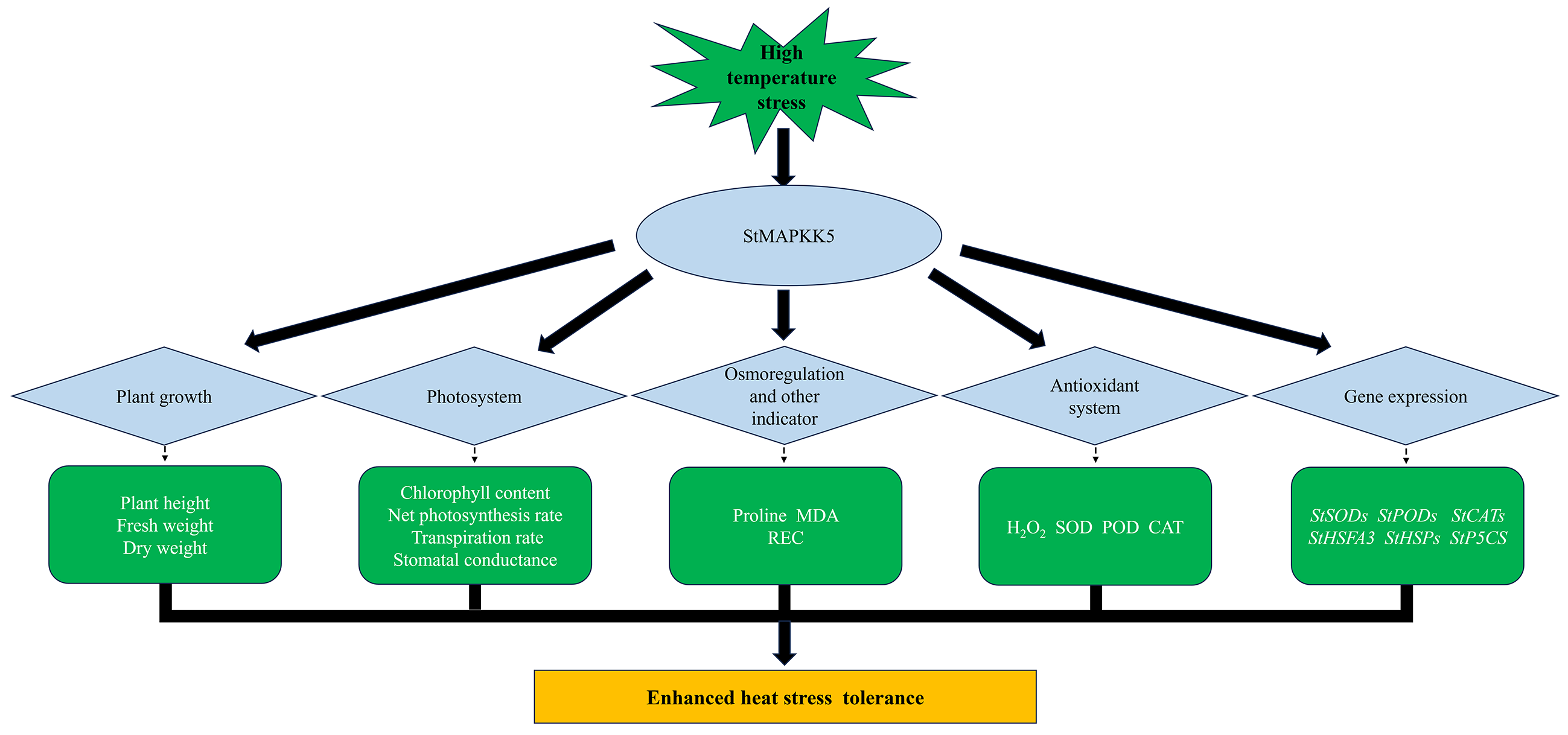

Supplement: Supplementary file 2 [file Image_1.tif]
